# Betalains in Some Species of the Amaranthaceae Family: A Review

**DOI:** 10.3390/antiox7040053

**Published:** 2018-04-04

**Authors:** Maria Graça Miguel

**Affiliations:** Faculdade de Ciências e Tecnologia, MeditBio, Universidade do Algarve, Campus de Gambelas, 8005-139 Faro, Portugal; mgmiguel@ualg.pt; Tel.: +3512-8980-0100

**Keywords:** betalains, alternanthera, amaranthus, beta, chenopodium, celosia, gomphrena

## Abstract

Natural pigments are largely distributed in the plant kingdom. They belong to diverse groups, with distinct biochemical pathways. Betalains with colours that range from yellow to red-violet can de divided into two main subgroups: betaxanthins and betacyanins. These types of pigments are confined into 13 families of the order Caryophyllales and in some genera of higher fungi (*Amanita muscaria*, *Hygrocybe* and *Hygrophorus*). The Amaranthaceae family includes diverse genera in which betalains are present: *Alternanthera*, *Amaranthus*, *Beta*, *Chenopodium*, *Celosia* and *Gomphrena*. The biosynthesis of betalains and their general biological properties were reviwed in the present work. In addition, the types of betalains present in some species of the aforementioned genera, their stability and production, as well as biological attributes, were reviewed.

## 1. Introduction

The colour of flowers, fruits, vegetables and grains result from the presence of chlorophylls, carotenoids, flavonoids and of the water-soluble anthocyanins and betalains [1,2]. Anthocyanins and betalains have never been detected jointly in plant tissues. Anthocyanin pigments (orange, red, purple, blue) occur in all members of the Angiospermae, whereas betalains occur only in 13 families of the order Caryophyllales [3,4].

Betalains are water-soluble pigments classified into betacyanins (red colour) and betaxanthins (yellow colour). They are dissolved inside the vacuoles as *bis*-anions. Betalains can be found in edible fruits and roots, flowers, stems and bracts of species belonging to the families of Caryophyllales, except for Caryophyllaceae and Molluginaceae [5]. These pigments have also been detected in some genera of higher fungi (*Amanita muscaria*, *Hygrocybe* and *Hygrophorus*) [6].

The present work aims to make a review of the betalains present in some species of the genera *Alternanthera*, *Amaranthus*, *Beta*, *Chenopodium*, *Celosia* and *Gomphrena*, which belong to the *Amaranthaceae* family.

## 2. Betalains

### 2.1. General Chemical Aspects of Betalains

**Figure 1:**
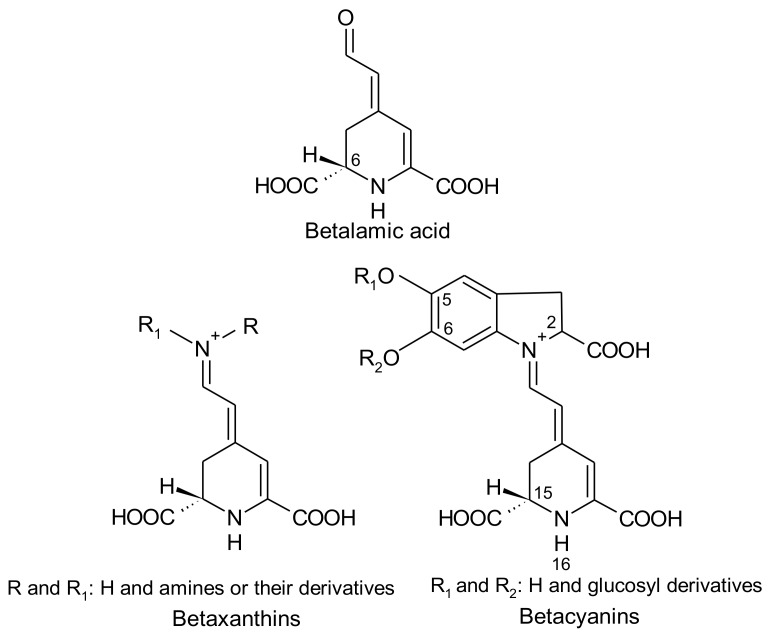
Structures of betalamic acid, betaxanthins and betalains.

Betalains are immonium derivatives of betalamic acid [4-(2-oxoethylidene)-1,2,3,4-tetrahydropyridine-2,6-dicarboxylic acid]. The condensation of this structure with amines and/or their derivatives generates yellow betaxanthins (dLatin: beta = beet; Greek: xanthos = yellow), whereas the condensation of betalamic acid with *cyclo*-dopa [*cyclo*-3-(3,4-dihydroxyphenylalanine)] or its glucosyl derivatives gives rise to violet betacyanins (Latin: beta = beet; Greek: kyaneos = blue) (Figure 1) [7,8]. The resulting glycosides of betacyanins can be linked to acylation groups, leading to several structures [9].

**Figure 2:**
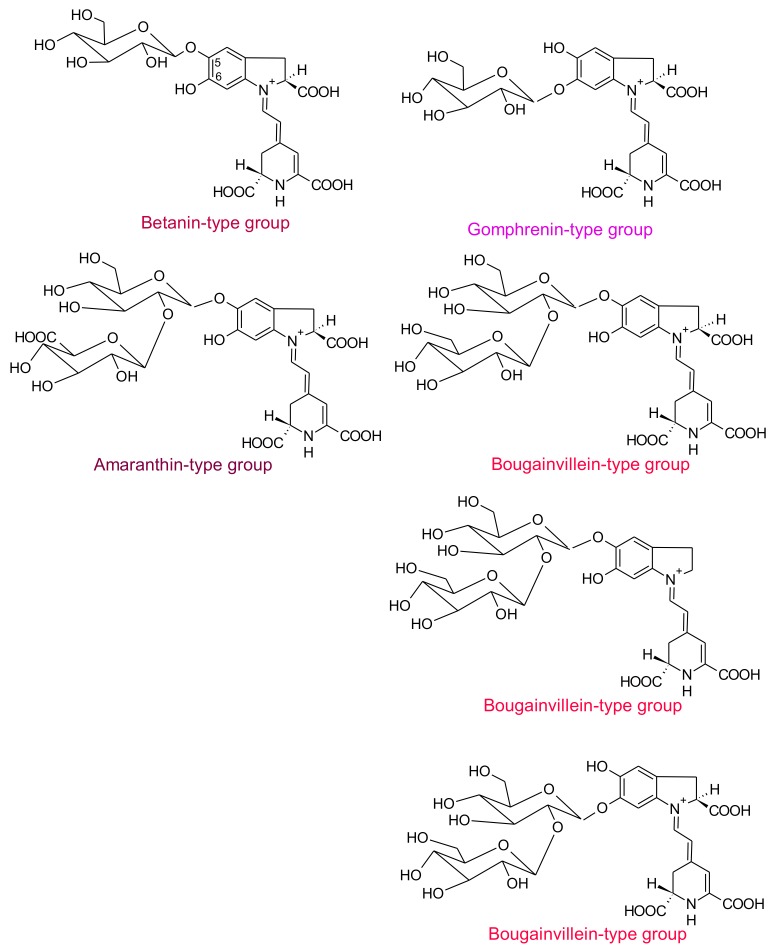
Example of structures of the different types of betacyanins.

Four structural types of betacyanins have been reported: betanin, gomphrenin, amaranthin and bougainvillein [6]. These structures differ by the attachment of glucosyl groups to the oxygen atoms in the *o*-position on the *cyclo*-dopa moiety [6]. Betanin-type group has a hydroxyl attached to the C_6_ carbon and a glucosyl or derivative on the C_5_ carbon (Figure 2), whereas gomphrenin-type group possesses a hydroxyl attached to the C_5_ carbon and a glucosyl or derivative on the C_6_ carbon (Figure 2). Amaranthin-type group has the glucuronylglucosyl moiety or derivative linked to the C_5_ carbon. Bougainvillein-type group may possess a diglucosyl moiety or derivative linked to the C_5_ carbon or to the C_6_ carbon of carboxylated or decarboxylated betacyanins [6]. Betaxanthins are divided into two groups: amino acid-derived conjugate group and amine-derived conjugate group. In both cases, they are linked to the conjugated moiety of betalamic acid [10].

The 1,7-diazaheptamethinium resonance system with conjugated double bonds of betalamic acid (Figure 1) is responsible for its lemon-yellow colour [7]. The conjugation of a substituted aromatic nucleus to this chromophore shifts the absorbance maximum from 480 nm in yellow betaxanthins to 540 nm in red purple betacyanins [1]. In addition, betacyanins display other absorption maxima: 270–280 nm due to the *cyclo*-dopa structure. Acylation with hydroxycinnamic acids generates a third maximum at about 300–330 nm. The addition of aliphatic acid derivatives did not affect the absorption. Decarboxylation at C-2 produces a slight hypsochromic shift of about 5 nm [7].

The absorption maximum of the betacyanins is influenced by the introduction of substituents in the backbone of the molecule. For example, glycosylation of betanidin (violet-red) at C_5_ gives rise to betanin (magenta). Generally, the introduction of a glucosyl group at C_5_ comes along with a hypsochromic shift of about 6 nm of the resulting betacyanin. The introduction of the same group at C_6_ brings about a greater bathochromic shift than 5-glycosylation. A second sugar moiety attached to the first apparently did not affect the spectroscopic properties [2,7]. Acylation with aromatic acids leads to a bathochromic shift up to 12 nm, whereas esterification with aliphatic acyl moieties only slightly or did not alter the maximum absorption of betacyanins [2,7].

As for betacyanins, in betaxanthins, structural modifications in the backbone may produce hypso- or bathochromic shifts. For instance, amine conjugates exhibit a lower absorption maximum than their respective amino acid counterparts [11].

Betaxanthins are fluorescent pigments which present maximum excitation wavelengths between 320 and 475 nm that correspond to blue light and emission maxima between 506 and 660 nm that correspond to green light. The fluorescence of betacyanins is weaker than that of betaxanthins but the intensity is enhanced by carboxyl groups or reduced by aromatic ring and hydroxyl groups [6,12].

### 2.2. Stability of Betalains

Despite betalains pigments are considered more stable than anthocyanins [13] there are several factors that negatively affect their stability: pH < 3 or pH > 7; high a_w_ (water activity); metal cations (Fe^3+^, Fe^2+^, Cu^2+^, Cu^+^, Sn^2+^, Al^3+^, Hg^2+^, Cr^3+^); high temperature; light; O_2_; H_2_O_2_, low pigment content; among other factors [7]. For these reasons, betalains have had little application in food industry as colourants, unless if they can be used in frozen foods, low temperature dairy products or short-shelf life foods [14].

**Figure 3:**
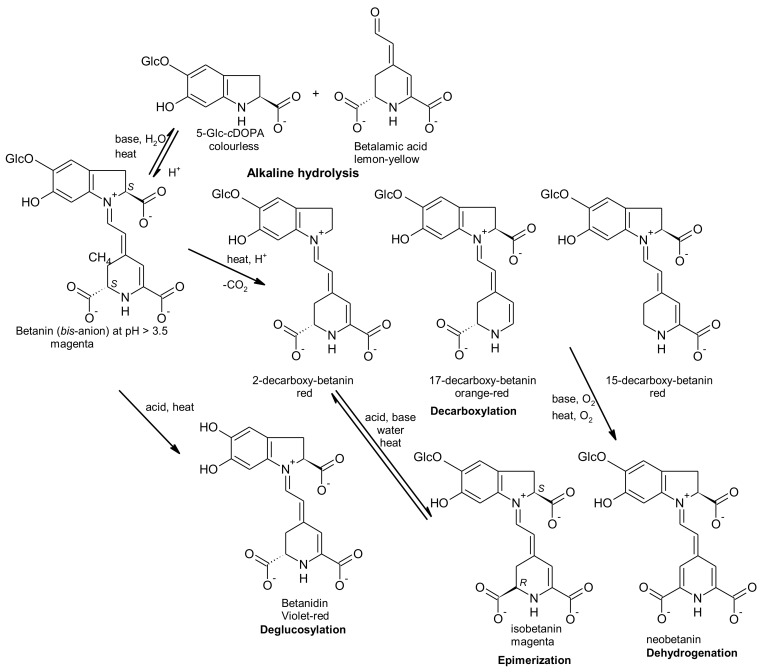
Betanin degradation products and some degradation pathways (adapted from [16]).

Heat mostly induces dehydrogenation in the presence of oxygen (oxidation), aldimine bond hydrolysis and decarboxylation of batalains, which induce a colour change to orange-yellow. At pH values < 3, the betalain structure is converted from anionic form (red) to cationic form (violet), generating a visible colour change from red to blue-violet shade. At pH values > 7, there is the aldimine bond hydrolysis of betalain, giving rise to betalamic acid and *cyclo*-dopa-5-*O*-glucoside and the colour change to yellow-brown. Metal ions can accelerate oxidation of betalains with the consequent loss of colours [15]. Epimerization is another process that can occur [16]. Figure 3 represents the possible chemical processes that can occur in betanin when exposed to acid or basic conditions or when submitted to heating, according to that previously reported by Gonçalves et al. [16].

### 2.3. Extraction, Separation, Identification and Quantification of Betalains

Betalains are generally extracted from ground plant material by maceration extraction with water, cold, or at room temperature or Soxhlet extraction. For a complete extraction, it is preferable to use methanol or ethanol solutions (20–50%, *v*/*v*). Before extraction, a short heat treatment (70 °C, 20 min) can be made for inactivating degradative enzymes, despite the possible destruction of some pigments. A slight acidification with HCl or citric acid or acidified ethanol (0.4 to 1% HCl) can be used for the precipitation of betacyanins. The addition of aqueous ethanol (95%) will give betaxanthins [1]. However, other techniques have been assayed to extract betalains, particularly from red beet, in order to enhance the yields in a shorter time period, to increase the cell membrane permeabilisation and to be more environmentally friendly—diffusion extraction, ultrafiltration and reverse osmosis and loose reverse osmosis (LRO), cryogenic freezing, aqueous two-phase extraction, pulsed electric fields and gamma irradiation, microwave and ultrasound-assisted extractions [11,17,18,19].

Purification and isolation of betalains is needed before their qualitative and quantitative analysis for eliminating possible interfering compounds. Chromatographic and electrophoretic procedures have been used to separate and/or purify betalains. For the separation of these pigments, ion chromatography on cation exchange resins followed by adsorption chromatography on polyamide column or a sequential chromatography on a series of Sephadex ion exchangers can be used [20]. Separation, identification and quantification of betalains have been carried through high performance liquid chromatography (HPLC) coupled to ultraviolet-visible (UV-Vis), photodiode array (PDA) mass spectrometry (MS) and nuclear magnetic resonance detection (NMR) by using reversed phase columns and binary gradient dilutions (acidified water with formic acid 2–5%, *v*/*v*) and acetonitrile or methanol. As betaxanthins are fluorescent pigments, the detection of these pigments may be done using fluorescence methods [20].

### 2.4. Biosynthesis of Betalains

**Figure 4:**
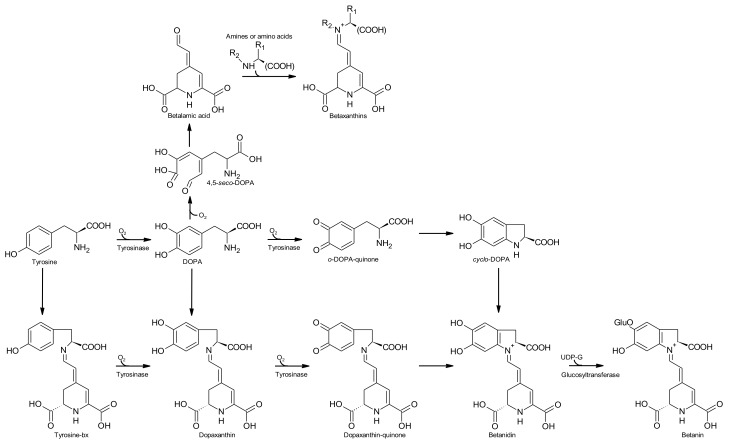
Simple scheme for the biosynthetic pathway of betalains (adapted from [21]).

The biosynthesis of betalains will be described according to the review by Gandía-Herrero and García-Carmona [21]. The pathways are schematised in Figure 4.

Betalains are secondary metabolites derived from the amino acid l-tyrosine. The amino acid undergoes hydroxylation and l-dopa is formed—the reaction is catalysed by the enzyme tyrosinase (or polyphenoloxidase). A second enzyme (4,5-dopa extradiol dioxygenase) catalyses the cleavage of dopa to form the intermediate 4,5-*seco*-dopa. A spontaneous intramolecular condensation occurs between the amine group and the aldehyde group of 4,5-*seco*-dopa, giving rise to betalamic acid (Figure 4).

Betalamic acid, a structure that holds the chromogenic electron resonance, may accept spontaneously amines or amino acids, which leads, by the reaction between the amine group of the amine and the aldehyde group of betalamic acid, to the formation of yellow betaxanthins.

For the formation of betacyanins two pathways could be considered:

Pathway 1. Formation of betanidin through the condensation with free l-dopa derivatives

l-Dopa, in the absence of a reducing agent (ascorbic acid or an analogous reducing agent), is transformed by tyrosinase to *o*-dopa-quinone in the presence of molecular oxygen. Then, a spontaneous cyclization takes place involving the amine group of the *o*-quinone that, through an intramolecular nucleophilic attack on the ring, forms the molecule *leuko*-dopa-chrome, also known as *cyclo*-dopa. This compound may react with betalamic acid to generate the pigment betanidin. This pigment is an intermediate in the formation of betacyanins. In the absence of a reducing agent, *cyclo*-dopa (unstable) undergoes a spontaneous oxidation with the formation of dopa-chrome with the simultaneous reduction of a molecule of dopa-quinone back to l-dopa. This mechanism was reported by Schliemann et al. [22], nevertheless is considered improbable to occur at a biological level [21].

Pathway 2. Formation of betanidin through tyrosine-betaxanthin (tyrosine-bx) and dopa-bx (dopaxanthin)

The reaction starts with the condensation of l-tyrosine with betalamic acid originating the pigment tyrosine-bx. This pigment is the substrate for tyrosinase that converts it in dopa-bx. This pigment is also substrate for tyrosinase that transforms it in dopaxanthin-quinone. In the absence of a reducing agent, this quinone undergoes an intramolecular nucleophilic attack with a consequent cyclization and formation of betanidin. Another possibility is the condensation of betalamic acid with l-dopa to form dopaxanthin [21].

Betanidin can incorporate a glucose residue in position 5, reaction catalysed by the enzyme betanidin-5-*O*-glucosyltransferase and betanin is formed. Other reactions of glycosylation and acylations may occur that lead to a wide variety of known betacyanins [21].

### 2.5. Role of Betalains in Plants

The function of betalains in plants is diverse: for plant propagation through pollen transfer or the dispersion of indigestible seeds by animals that are attracted by the colour of flowers and fruits; as a repelling signal to deter herbivores; to screen tissue against damaging ultraviolet (UV) radiation; for increasing pathogen resistance and improving viral defence; for providing higher tolerance to heat stress; as osmoregulators by modelling the amino acid pool from betaxanthins, among other functions [2,23,24,25].

### 2.6. Importance of Betalains for Consumers

Some species are edible sources of betalains: red beet roots (*Beta vulgaris* subsp. *vulgaris* convar. *vulgaris* var. *vulgaris*; Conditiva Group); fruits of cacti, particularly *Opuntia ficus-indica*; the dragon fruits from *Hylocereus polyrhizus*; Swiss chard (*Beta vulgaris* subsp. *vulgaris* convar. *cicla*. var. *flavescens*; Flavescens Group). For instance, the extracts of beetroot containing betalains are pink or violet pigments used as food additive under the E-162 code (betanin), in the European Union, or 73.40 of the Food and Drug Administration (FDA) [5]. E-162 is mainly applied for colouring fruit yogurts, ice-creams, jams, chewing gums, sauces and soups. The same pigment has also been used in cosmetics and pharmaceuticals [26].

Beyond the application of betalains as colourants in food industry, these secondary metabolites also possess biological properties beneficial for human health: anti-inflammatory, antioxidant, inhibitory effect towards skin and lung cancer in mice and chemo-preventive properties (positive effects on metabolic, cardiovascular and gastrointestinal health in humans), with the advantage to be devoid of toxicity, mutagenic action or allergic reactions [2,5,27,28,29,30,31,32,33,34].

The colouring properties of betalains along with their biological properties have led to the development of in vitro strategies for producing these secondary metabolites, however this has been without success. Georgiev et al. [35] reviewed progress in plant in vitro systems for producing betalains. At the same time, the authors cited the factors that could be manipulated for improving the betalain yields as well as the bioreactor systems that could be used.

### 2.7. Production of Betalains

Owing to the importance of betalains as natural pigments, there has been research with the aim to obtain them by synthetic procedures. Generally, it starts with the production of betalamic acid or even better, with extraction in ethyl acetate from natural sources and then condensation with amines or amino acids. Nevertheless, this procedure gives rise to very low yields. Another way to produce betalains is based on a combined procedure of betanin degradation and betalains synthesis. This methodology requires an immediate purification to avoid reverse reactions [36,37].

New methods have been developed and one of them consists of betalamic acid derivatized by a support generated from a primary amine polymer. This material is stable for months. The imine bond between polymer surface and betalamic acid may be broken at mild conditions through the addition of amines in water over a pH range at room temperature. Betalains (betaxanthins and betalains) originate from the displacement of betalamic acid from the surface polymer and its reaction with amines or amino acids. This procedure is optimized for the formation of betalains derived from tyramine, dopamine, pyrrolidine and indoline [38].

Plant cell and tissue cultures have also been used to produce betalains because such production is independent on the climate or soil properties, pest and diseases; it also allows continuous supplies with uniform quality and yield. However, the yield production is generally low and almost always needing bioreactor systems for improving large scale production [35]. Some examples include the production of betalains by hairy root cultures, cell suspension and callus culture of beetroot [39,40,41,42,43,44,45,46]. Generally, in these works, the authors used methods and studied factors that could improve the production or recovery of betalains by cell cultures of *Beta vulgaris* (radiation, light, utilization of adsorbents, changes in the composition of the culture medium, alterations of the bioreactors, among other factors). Other approaches include improving betalain yield in beet through the improvement of the utilization of tyrosine or enhancement of its production, depending on the beet genotype [47] or developing new betalains sources (microbes and/or plants) by metabolic engineering [48].

### 2.8. Biological Properties of Betalains

The antioxidant activity of betalains is well documented in several works and using diverse methodologies, e.g., scavenging of the 2,2′-azino-bis-(3-ethyl-benzthiazoline-6-sulfonic acid) (ABTS^●+^) and 1,1-diphenyl-2-picrylhydrazyl (DPPH) free radicals [49]. The relationship between the chemical structure of betalains and the activity was also investigated and different pigments exhibited diverse capacity for scavenging free radicals. The tested betalains decreased its scavenging capacity in the following order: simple gomphrenins > acylated gomphrenins > dopaminebetaxanthin > (*S*)-tryptophan-betaxanthin > 3-methoxytyraminebetaxanthin > betanin/isobetanin > celosianins > iresinins > amaranthine/isoamaranthine. Betalains, particularly red-violet gomphrenin type betacyanins and yellow betaxanthins, had strong antioxidant activity when compared to the traditional standards (ascorbic acid, rutin and catechin). There is an impact of the chemical structure of betalains on the antioxidant activity: the capacity for scavenging free radicals increased with the number of hydroxyl groups and imino groups and decreased with more glycosylation of aglycones in the molecule. The capacity for scavenging other free radicals, such as peroxyl (ROO•) free radicals or nitric oxide (NO•), by some betacyanins was also dependent on the number of hydroxyls. For example, betanin was less effective than betanidin [50,51]. Additive effects were observed when betanin or betanidin were blended with α-tocopherol and incorporated in liposomes [51]. However, and according to some authors [52], the antioxidant activity of betalains, particularly betaxanthins, is not linked to the presence of hydroxyl groups or aromaticity of the pigment structure. According to the same authors there is an ‘intrinsic activity’ of betaxanthins which might be associated with the common resonance system supported by the two nitrogen atoms of the betaxanthin. Nevertheless, the presence of hydroxyl groups in betaxanthins increase their ABTS free radicals scavenging activity [52].

The capacity for scavenging ABTS•^+^ of betalamic acid was better when compared to that of trolox. The same pigment was also able to reduce Fe^3+^ to Fe^2+^. These activities were attributed to the extended conjugated system of betalamic acid, nevertheless dependent on the pH of the environment: pH values above 5.5 increased the activity of betalamic acid [53]. The importance of the pH on the activity was also reported for betanin, the main pigment of red beet [28] and other betalains [12].

The oxidation mechanisms of betanin and some of its derivatives (2-decarboxybetanin, 17-decarboxybetanin, 2,17-bidecarboxybetanin and neobetanin) in the presence of ABTS and the intermediates that are generated during the reactions, was studied by Wybraniec et al. [54].

Betanin and betanidin were able to prevent lipid peroxidation in microemulsions, membranes and low-density lipoprotein (LDL) catalysed by cytochrome c, H_2_O_2_-activated metmyoglobin, iron redox cycle and lipoxygenase [55]. Betanin could also inhibit 71% lipid peroxidation in a liposome system [56]. The protection of LDL oxidation in vivo by betalains was attributed to the transactivation of paraoxonase 1 (PON1), an antioxidant enzyme produced in the liver [57].

*Beta vulgaris* extract was able to inhibit protein oxidation induced by hypochlorous acid released from active neutrophils. This property is beneficial on pain associated with osteoarthritis [58].

Lipoxygenase (LOX) and cyclooxygenase (COX) are enzymes that are involved in the conversion of arachidonic acid to inflammatory mediators leukotrienes and prostaglandins, which are inflammatory mediators. Betanidin and betanin were able to inhibit soybean LOX, being this activity higher than catechin (control) [55]. Betanin was able to inhibit COX-1 and COX-2 [56]. Betaxanthins had also capacity for inhibiting COX, phenethylamine-bx was a potent inhibitor of this enzyme by interaction with Tyr-385 and Ser-530 residues close to the active site of COX [34]. The anti-inflammatory activity of betalains may also be due to their action against cellular mediators of inflammation, such as intercellular cell adhesion molecule-1 (ICAM-1) significantly repressed by betanin [59].

In different studies, betanin and betalain-rich red beet extract induced phase II enzyme quinine reductase [60,61]. This activity is related to the hepatoprotective effect of these pigments: Feeding rats with betalain-rich red beet juice diminished the action of the hepatotoxic compounds *N*-nitrosodiethylamine, carbon tetrachloride and 7,12-dimethylbenz(a)anthracene, due to the improved antioxidant status and increased expression of quinine reductase [62,63]. Moreover, administration of *Amaranthum tricolor* extracts containing betalains to diabetic rats significantly reduced blood cholesterol, triglycerides and LDL (low density lipoproteins) level and increased the high-density lipoproteins level (HDL), having thus the potential to prevent cardiovascular disorders [64,65]. Betalains from red beet juice and chips inhibited neutrophil oxidative metabolism in obese individuals [66], suggesting that betalains may have an interesting role in the management of hyperlipidaemia [64]. The antimalarial activity of betanin/amaranthin-rich extract of *Amaranthus spinosus* in mice was due to the capacity of these pigments for chelating the inner cations Ca^2+^, Fe^2+^ and Mg^2+^ and for blocking the choline intracellular transport of parasites [30].

Beet root pomace and betalains were able to reduce the growth of several Gram-positive and Gram-negative microorganisms, nevertheless the mechanisms involved in the microbial inhibition is scarce [64]. Other attributes have been found in vitro and in vivo for betalains such as anti-diabetic, anti-tumoural, regulator of metabolic dysfunctions, among other properties that are compiled in three recent reviews [5,14,64].

## 3. Amaranthaceae

*Amaranthaceae* family is characterised by its diversity in secondary metabolites: essential oils, sesquiterpenes, diterpenes, triterpenes, phenolic acids, flavonoids and betalains [67]. This family includes diverse genera in which betalains have been reported: *Alternanthera*, *Amaranthus*, *Beta*, *Chenopodium*, *Celosia* and *Gomphrena* [27,29,68,69,70].

Several properties have been attributed to some of these genera: antioxidant, positive effects on metabolic, cardiovascular and gastrointestinal health in humans; antimalarial, inhibitors of LOX and COX [27,28,29,30,33,34,49,70].

### 3.1. Gomphrena globosa *L.*

*Gomphrena globosa* L. (globe amaranth) is native to Panama and Guatemala and presents some resistance to drought and high adaption ability to poor soils or colder lands [71]. It is an ornamental plant largely cultivated in mild climatic regions [72]. Leaves and inflorescences’ decoctions and other parts of globe amaranth are used in folk medicine in the treatment of some inflammatory respiratory diseases, such as bronchial asthma, acute and chronic bronchitis or whooping cough; jaundice; hypertension; some kidney problems such as gall stones; prostate problems; diabetes; and to stop local haemorrhage [71,73,74,75].

**Figure 5:**
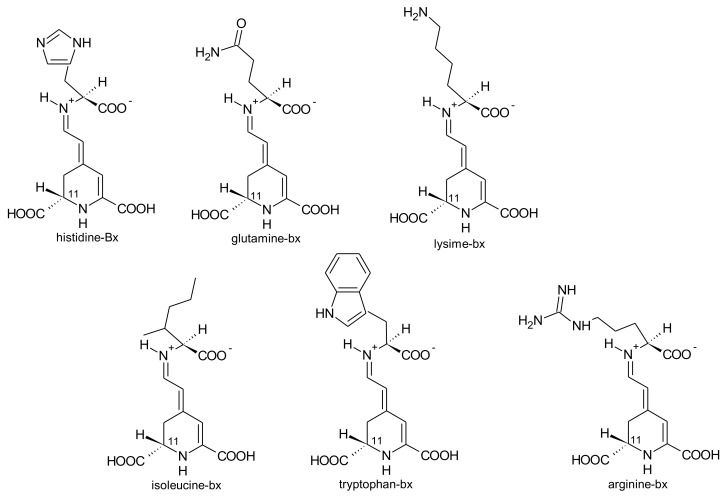
Betaxanthins found in red inflorescences of *G. globosa* L.

**Figure 6:**
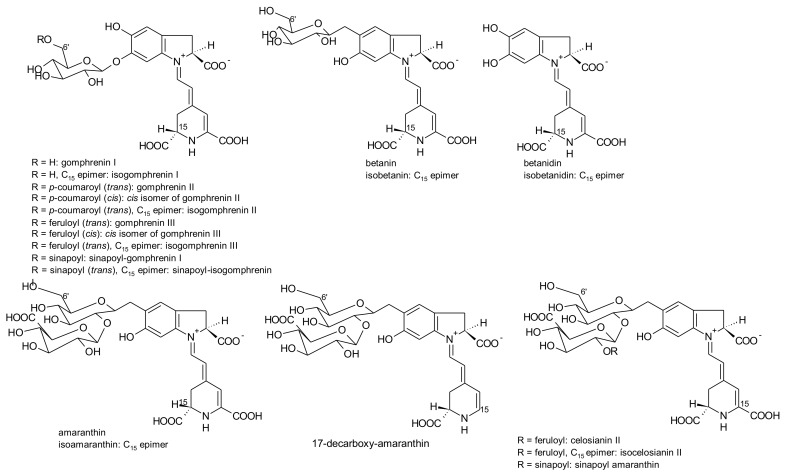
Betacyanins found in *G. globosa* L. inflorescences and *Amaranthus*.

*G. globosa* inflorescences can have purple-, red- and orange colour [76]. According to the variety, the levels of betacyanins and betaxanthins changed. Purple inflorescences had the highest amounts of betacyanins (556.8 mg/kg, fresh weight) and only traces of betaxanthins. The lowest concentrations of betacyanins were found in orange varieties (30.6 mg/kg, fresh weight), whereas the red variety had the highest concentrations of betaxanthins (75.0 mg/kg, fresh weight) [76]. For the first time, betaxanthins histidine-bx (muscaarin VII), arginine-bx, glutamine-bx (vulgaxanthin I), lysine-bx, isoleucine-bx and tryptophan-bx (Figure 5) were reported in the red petals of globe amaranth [76]. The betacyanins identified in the red and purple petals of globe amaranth were amaranthin, betanin, gomphrenin I, gomphrenin II, gomphrenin III, isogomphrenin I, isogomphrenin II, isogomphrenin III, isoamaranthin, isobetanin, betanidin, isobetanidin, celosianin II and isocelosianin II [76,77]. In addition, six more betacyanins were tentatively identified [76]: 17-decarboxy-amaranthin, sinapoyl-amaranthin, *cis*-isomer of gomphrenin III, *cis*-isomer of gomphrenin II, sinapoyl-gomphrenin I and sinapoyl-isogomphrenin I (Figure 6).

17-Decarboxy-amaranthin, 17-decarboxy-isoamaranthine, sinapoyl-gomphrenin I, amaranthin, isoamaranthin, betanin, isobetanin, gomphrenin I, gomphrenin II, gomphrenin III, isogomphrenin II, isogomphrenin III, isogomphrenin I, celosianin II and isocelosianin II were found in extracts of red inflorescences of *G. globosa* [49,72,78,79,80,81,82,83].

The type of extraction and extraction solvent used for obtaining betacyanins from *G. globosa* inflorescences gave rise to quantitative differences in the betacyanins identified by Silva et al. [71]. Maceration of inflorescences with methanol for 30 min, at room temperature, with agitation (400 rpm) revealed to be the most effective extraction, capable to extract higher amounts of betacyanins (gomphrenin I, gomphrenin II, gomphrenin III, isogomphrenin I, isogomphrenin II, isogomphrenin III, sinapoyl-gomphrenin I and isosinapoyl-gomphrenin I), being isogomphrenin III the most abundant (1.25 mg/g) [71]. The same components have been already reported by Ferreres et al. [80] in aqueous extracts of *G. globosa* inflorescences obtained by maceration for 30 min at room temperature, in obscurity and with agitation (400 rpm).

In order to obtain the maximal amount of betacyanins, Roriz et al. [81] used the response surface methodology (RSM), with which it is possible to optimize the maceration conditions simultaneously (time, temperature, ethanol-water proportion and solid-liquid ratio). The betacyanins identified were gomphrenin and isogomphrenin II and III and the best conditions of extraction for obtaining the highest pigment content (~45 mg/g) were: 165 min, 25 °C, 0% ethanol and 5 g/L of solid-liquid ratio. The best extraction method of gomphrenin and isogomphrenin II and III from flowers, bract and bracteoles of *G. globosa* was ultrasound assisted extraction in the following conditions: time = 22 min; power = 500%; Ethanol = 0%; and ratio solid/liquid = 5 g/L [82].

Matrix-assisted laser desorption/ionization quadrupole ion trap time-of-flight mass spectrometry (MALDI-QIT-TOF-MS) was a method of identification betacyanins of crude extracts from *G. globosa* without any previous purification [72]. The identification by this method revealed to be rapid without any previous purification of extracts but presented some limitations, including the difficulty to distinguish the isomers of the betacyanins, which needs an additional analysis by HPLC or LC-MS [72].

Extracts of *G. globosa* had antioxidant activity, through the capacity for scavenging diverse free radicals (DPPH, superoxide, nitric oxide) and anti-inflammatory activity since they were able to decrease the nitric oxide content in the supernatant of the lipopolysaccharide (LPS)-stimulated RAW 264.7 macrophages culture [71]. However, the authors were unable to attribute these activities only to the betalains present in the extracts. Other components had also a role on the activities detected.

Roriz et al. [79] studied the antioxidant activity of three extracts (*Pterospartum tridentatum* (L.) Willk. *Gomphrena globosa* L. and *Cymbopogon citratus* (DC) Stapf.), using four methods (DPPH scavenging activity, reducing power and inhibition of lipid peroxidation by β-carotene bleaching inhibition and TBARS assays). They found that *G. globosa* extract, the sole possessing betalains together with the lowest amounts of phenols, had the worst activity. Therefore and according to the authors, the antioxidant activity found in samples was highly dependent on the profile in phenolic compounds and their amounts.

### 3.2. Alternanthera

The *Alternanthera* genus includes 80 species. Many species of *Alternanthera* have been used in folk medicine for the treatment of infections, genital inflammation and fever [84,85,86]. *Alternanthera brasiliana* (L.) Kuntze and *Alternanthera tenella* Colla are native to tropical and sub-tropical regions of Australia and South America [69].

In Brazil, the leaves of *A. brasiliana* (Brazilian joyweed) have been used in folk medicine as galactogogue, cholagogue and a diuretic for the treatment of indigestion and abortifacient [87,88]. In Peru, whole plant is used to treat bronchitis and asthma. Laboratorial studies have demonstrated that the extracts of *A. brasiliana* possess modest antioxidant activity; anti-inflammatory; wound healing; analgesic, antiviral and antitumor activities [88,89,90,91,92,93]. These findings were generally attributed to the phenols, terpenes and sterols present in the leaves and not to their pigments or betalains. Such does not mean the absence of activity of these secondary metabolites, only very few works can be found that aimed to study the betalains of leaves from *A. brasiliana* [69]. The antimicrobial activity of *A. brasiliana* extracts on several microorganisms (*Staphylococcus aureus*, *S. epidermidis*, *Escherichia coli*, *Bacillus subtilis*, *Micrococcus luteus*, *Candida albicans* and *Saccharomyces cerevisiae*) was weak [94,95].

*Alternanthera tenella* Colla (joyweed) is a weed that occurs in crops and uncultivated areas in northwest Brazil but also in other regions, like the state of São Paulo [85,96,97]. *A. tenella* has been used in popular medicine to treat the infections, fevers, bruises and itches [98,99]. The plant has been also used as diuretic [100,101]. Laboratorial experiments have revealed that *A. tenella* extracts have immunomodulatory, antioxidant, anti-hyperalgesic, antimicrobial and antiparasitic properties [85,96,101,102,103,104]. As reported for *A. brasiliana*, these activities have been generally attributed to the phenols, particularly flavonoids. However, the possible role of betalains in such activities was not evaluated by the authors. In some locations of India there are diverse tribes that use the leaves of *A. brasiliana* and *A. tenella* as vegetables that can be consumed fresh or cooked or even fried in diverse ways, along with rice [69,105,106].

**Figure 7:**
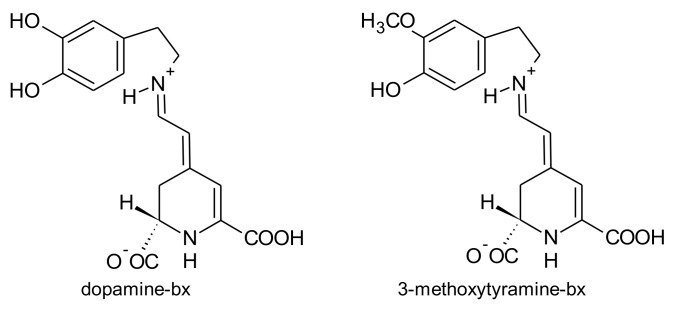
Betaxanthins found in Alternanthera brasiliana and *Alternanthera tenella*.

*A. brasiliana* and *A. tenella* leaves and stems are strongly coloured. Betalains from these parts of both species were extracted by Deladino et al. [69]. In both species, leaves had higher amounts of total betalains than the respective stems. The highest amounts of betalains were found in the leaf extracts of *A. brasiliana* (89.40 μg/g, dry weight), while the leaf extracts of *A. tenella* only had 17.29 μg/g (dry weight) [69]. Amaranthin, isoamaranthin, betanin and isobetanin (Figure 6) were quantified in the leaf and stems extracts of *A. brasiliana* and *A. tenella*. In all samples, amaranthin predominated. Dopamine-bx and 3-methoxytyramine-bx (Figure 7) were the sole betaxanthins tentatively identified in the same extracts but in very small amounts [69].

The antioxidant activity, measured trough the ferric reducing antioxidant power, was evaluated in leaf and stems’ extracts of *A. brasiliana* and *A. tenella*. Leaf extracts of *A. tenella* possessed the highest antioxidant activity, being coincident with the highest amounts of flavones found in this extract, particularly vitexin and its *C*-glycosides derivatives. The authors concluded that this activity could only be attributed to these flavones and not to betalains [69]. These results disagree with those reported by Rodrigues-Brandão [103], since the antioxidant activity was related to the betacyanins accumulated by *A. tenella* growing in vitro. Both increased with the addition of salicylic acid to the culture medium. In order to enhance the production of betalains, some authors assayed the action of tyrosine added to the in vitro cultures of *A. tenella* [98]. The authors reported a negative effect on in vitro growth of this species, affecting the formation of shoot and roots, nevertheless increased the biosynthesis of betacyanins, measured as total amaranthin. Higher production of betaxanthins and betacyanins was observed when *A. brasiliana* plants were submitted to blue and white light, whereas *A. tenella* plants did not show any significant differences in the amounts of these pigments accumulated when submitted to the same light types [102].

### 3.3. Amaranthus

The word *Amaranthus is* derived from the Greek word “Anthos” (Flower) which means everlasting, immortal [107,108]. *Amaranthus* known as amaranth is a genus of herbs that includes 60–70 species. Most of the species are annual weeds. Species of this genus can be a source of pseudo-cereals, vegetable and ornamentals. In Europe and America, *Amaranthus* are used as pseudo-cereals, whereas in Africa they are planted as vegetables [109,110].

Amaranth species were classified in vegetable *Amaranthus* (*A. tricolor* L.); grain *Amaranthus* (*A. hypochondriacus*, *A. caudatus*, *A. cruentus*); weed *Amaranthus* (*A. spinosus*, *A. viridis*, *A. retroflexus*, *A. graecizans*, *A. dubius* and *A. hybridus*) [109,110]. Vegetable *Amaranthus* possess inflorescences formed by mostly or exclusively axillary glomerules or with short spikes; whereas grain *Amaranthus* possesses an apical large to moderately large complex inflorescence with aggregates of cymes. Some weed species may present morphological similarity with leafy vegetable form and others with grain form of *Amaranthus* [109,110].

Despite that classification, some species may be used either as vegetable or grain. For example, leaves of the young *A. cruentus* plants may be used in salads and soups and the grains are utilized into breads, cakes, cookies, confectionary and soups [111]. Leaves of the young *A. tricolor* plants are used in salads and soups and the applications of *A. caudatus* grains are the same of those reported for *A. cruentus*. *Amaranthus spinosus* is not considered safe for consumption either by humans or livestock [111].

Grains of amaranth have been considered as possessing high nutritional value owing to their protein content, levels of essential amino acids (lysine and methionine) when compared to other grains. Amaranth grain is also considered a good source of insoluble fibre. Amaranth is also a rich source of soluble fibre, vitamins (riboflavin, niacin, ascorbic acid) and mineral contents (calcium and magnesium) [111]. Beyond these components, there are also secondary metabolites such as phenols and pigments (betacyanins) in the leaves of the vegetable amaranth *A. tricolor* as well as in the grain amaranth *A. cruentus* and *A. caudatus* [112]. Some genotypes of grain amaranth have much higher betacyanins over a longer growth period than vegetable amaranth species [113,114].

*Amaranthus tricolor* L. (Chinese spinach, tampala, Joseph’s coat), the main type of vegetable amaranth, is native to south or south-east Asia and then was dispersed all over the tropical and temperate regions [115]. This species adapts well to adverse growing conditions, is resistant to heat and drought, with the advantage to be rich in carotenoids, proteins, vitamins and dietary fibre [116]. This has made *A. tricolor* as an alternative source of nutrition for people living in developing countries with little access to protein-rich food [116,117]. This species has diverse leaf colours (red, purple) owing to the accumulation of various pigments, such as betacyanins [118].

*A. tricolor* was used as sudorific, febrifuge, emollient, blood purifier, on the treatment of menstrual disorders, eruptive fevers, pain, colic, anaemia, dysentery, skin diseases, dropsy, toothache, sore throat, cough, bronchitis, bilious disorder and diabetes [119,120]. Extracts of *A. tricolor* have been reported as antioxidant; hepatoprotetive; antimicrobial; antiproliferative on human AGS (gastric), CNS (central nervous system, SF-268), HCT-116 (colon), NCl-H460 (lung) and MCF-7 (breast) cancer cell lines; anti-inflammatory by inhibiting both cyclooxygenases isoenzymes (COX-1 and COX-2) and also by downregulating proinflammatory cytokine gene expression (TNF-α (tumour necrosis factor-α, IL-1(interleukin-1) and IL-6) in advanced glycation end products-induced cells; among other properties [120,121,122,123,124,125]. Generally, the authors related these activities with phenolic compounds including flavonoids.

Amaranthin and isoamarathin were extracted from seedlings and leaves of three genotypes of *A. tricolor* (Figure 6) [83,113]. Amaranthin was at higher concentrations. The authors found a relationship between betacyanins and proteins quantified in the samples [113]. Later on, [72] using MALDI-QIT-TOF-MS also identified amaranthin and isoamaranthin in the extracts of *A. tricolor* seedlings without previous purification.

As aforementioned, amaranthin (betanidin 5-*O*-β-glucuronosylglucoside) is the main betacyanin pigment of the vegetable amaranth. Betanidin glucosyltransferase is the enzyme that catalyses the transfer reaction of glucose molecule from UDP-glucose (uridine diphosphate-glucose) to betanidin. Betanidin glucosyltransferase has been isolated from several sources, for the first time from the leaves of *A. tricolor* by Das et al. [119]. In this work, the authors reported that this enzyme was able to catalyse the transfer of glucose to betanidin as well as to the flavonoids kaempferol and quercetin.

Total betaxanthins and betacyanins were quantified in the leaves of four cultivars of vegetable amaranth (Rocto alta, Rocto ranga, Alto pati, Baromashi), from Bangladesh [115]. Betaxanthins were in higher concentration than betacyanins in Alto pati, Baromashi cultivars. The amounts of both groups of pigments as well as the antioxidant activity were highly dependent on the cultivars [115]. The antioxidant property was related with the phenols and not with the betalains [115,126]. Differences in the amounts of betacyanins among cultivars were also previously reported by some authors [113,127,128]. Factors that may influence the accumulation of these pigments in the leaves of the vegetable amaranth include the growth stage (older plants had higher amounts of betacyanins); the position of the leaves within the shoot canopy (the apical leaves accumulated higher betacyanins than those of middle and basal leaves) [128]; photoperiod (12 h photoperiod revealed to be the best for the accumulation of betacyanins), which were coincident with the highest antioxidant activity found by the authors [129]; and food processing factors (low temperature, darkness and absence of oxidants maintained the stability of betalains of vegetable amaranth) [128].

The betacyanin accumulation in leaves of vegetable amaranth in response to natural climatic conditions (temperature, humidity and sunlight) was dependent on the cultivar [127]. This study permitted to separate the cultivars in different groups, according to the response to the natural climatic changes and may facilitate the selection and development of appropriate cultivars with desired traits in relation to the influence of climate [127].

Betacyanins’ accumulation by red amaranth cultivar Rocto alta can be affected by different coloured shade polyethylene, the highest betacyanins’ content occurred under blue colour polyethylene shade followed by yellow and white polyethylene shade, whereas green polyethylene shaded leaves exhibited the lowest amounts of betacyanins [118].

The biotechnological production of secondary metabolites is independent on the climate variations and soil properties, pests and diseases. In addition, such approach is also appropriate for studying the metabolic pathways. Biswas et al. [130] were able to obtain stable *callus* line of *A. tricolor* with enhanced production of betacyanins. Beyond the red-violet amaranthin and its C_15_-epimer isoamaranthin, the authors reported the production of new pigments belonging to the betaxanthins’ group: methyl derivative of arginine-bx and an unidentified betaxanthin. The production of betacyanins by seedlings obtained from *callus* cell line of *A. tricolor* was also reported and positively influenced by light and cytokinins [131]. The expression of five betalains’ biosynthesis genes was modulated by phytohormones but there are presumably also other factors that influence betalain production [132].

### 3.4. Beta vulgaris *L.*

There are four agriculturally groups within the subspecies *vulgaris* (cultivated beets): sugar beet (*B. vulgaris* ssp. *vulgaris* convar. *vulgaris* var. *altissima*), garden beet, table or red beet (*B. vulgaris* ssp. *vulgaris* convar. *vulgaris* var. *vulgaris*), fodder beet (*B. vulgaris* ssp. *vulgaris* convar. *vulgaris* var. *crassa*) and leaf beet, mangold, chard or silver beet (*B. vulgaris* ssp. *vulgaris* convar. *cicla*) [133].

The red beet is a traditional and popular vegetable in many parts of the world and has been also used to produce red juice and natural pigment in dairy products, beverages, candies and beef [134,135]. The red beetroot is the major commercially exploited betalain crop is red beetroot, being among the top ten vegetables known with the strongest antioxidant activity [11,136].

Over 80% of the total pigments of red beetroot are constituted by betacyanins, particularly betanin and its C_15_ isomer, isobetanin, whereas vulgaxanthin I is the predominant betaxanthin (yellow) [135,137]. 

Lee et al. [135] reported four main betalains in 9 cultivars of red beetroot, whose concentrations and proportions were cultivar dependent: betanin and isobetanin (betacyanins) and vulgaxantin I and miraxanthin V. Betacyanin:betaxanthin content ratio in red beetroot generally ranges about 1:3, depending on the cultivar and technology for obtaining juices [138].

Slatnar et al. [138] identified and quantified 15 betacyanins and 7 betaxanthins in red and yellow coloured cultivars grown in the field and at the same time compared the content of the analysed compounds in beetroot flesh, peel and petiole. The betacyanins identified included: 2′-*O*-glucosyl-betanin, betanin, 2′-*O*-glucosyl-isobetanin, isobetanin, isoprebetanin, betanidin, 2,17-bidecarboxy-neobetanin, 17-decarboxy-neobetanin, 2-decarboxy-neobetanin, neobetanin, 2,17-bidecarboxy-2,3-dehydro-neobetanin, 6′-*O*-feruloyl-2′-*O*-glucosyl-betanin, 6′-*O*-feruloyl-2′-*O*-glucosyl-isobetanin, 2-decarboxy-2,3-dehydro-neobetanin, 6′-*O*-feruloyl-betanin/isobetanin. The betaxanthins identified were: glutamine-bx (vulgaxanthin I), proline-isobx (isoindicaxanthin), proline-bx (indicaxanthin), valine-isobx, isoleucine-isobx, isoleucine-bx/leucine-isobx (isovulgaxanthin IV) and phenylalanine-isobx.

Not all identified compounds were present in all parts of the plant [138]. For example, betanin and 2-decarboxy-neobetanin were only detected in the peel. Others were absent in flesh: 2,17-bidecarboxy-2,3-dehydro-neobetanin, 6′-*O*-feruloyl-2′-*O*-glucosyl-betanin, 6′-*O*-feruloyl-2′-*O*-glucosyl-isobetanin and 6′-*O*-feruloyl-betanin/isobetanin. In red beetroot, only vulgaxanthin, isoleucine-isobx, isoleucine-bx/leucine-isobx (isovulgaxanthin IV) were present and only in flesh and peel.

More recently, Sawicki et al. [136] reported eighteen betacyanins and twelve betaxanthins in thirteen varieties and in the root parts of red beet, dominating betanin and isobetanin (betacyanins) and vulgaxanthin I (betaxanthins) as previously described [135,137]. The root peel had the highest betalain concentration.

**Figure 8:**
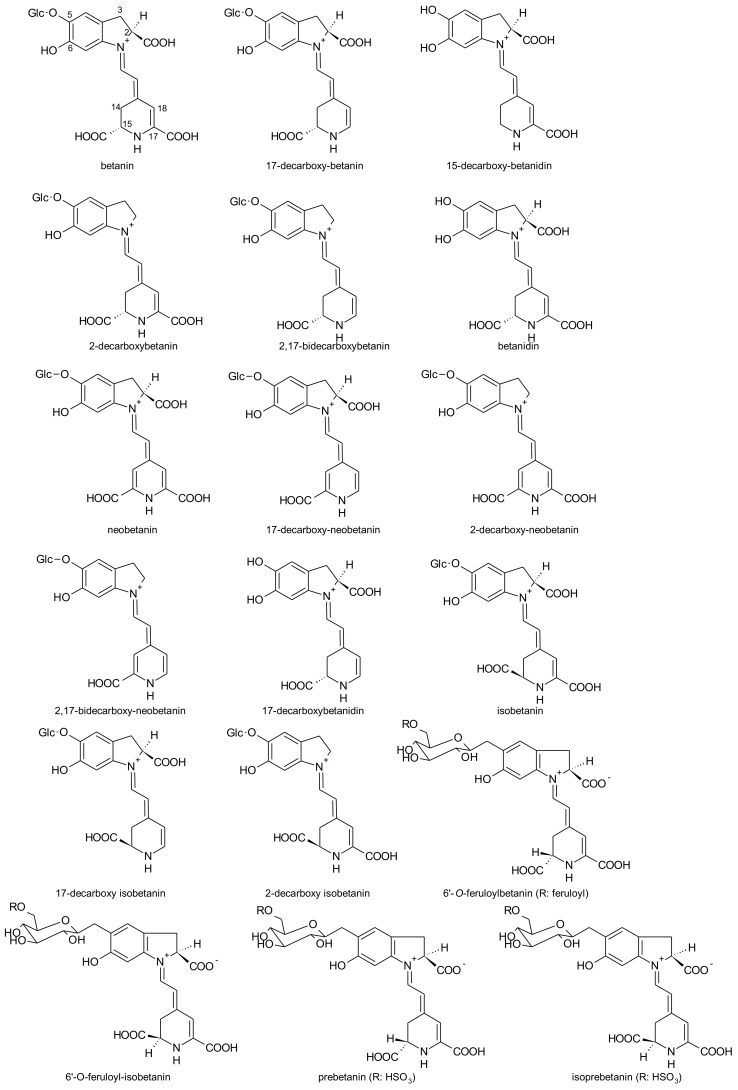
Betacyanins identified in the root parts of red beet.

According to Sawicki et al. [136], the betacyanins identified were: prebetanin, betanin, isoprebetanin, betanidin, isobetanin, 17-decarboxy-neobetanin, 17-decarboxy-betanidin/isobetanidin, neobetanin, 15-decarboxy-betanidin/isobetanidin, 17-decarboxy-betanin, 17-decarboxy-isobetanin, 2-decarboxy-neobetanin, 2,17-bidecarboxy-betanin/isobetanin, 2,17-bidecarboxy-neobetanin, 6′-*O*-feruloyl betanin, 6′-*O*-feruloyl-isobetanin, 2-decarboxy-isobetanin, 2-decarboxy-betanin (Figure 8). The betaxanthins identified were: glutamine-bx (vulgaxanthin I), valine-bx, leucine-bx (vulgaxanthin IV), asparagine-bx (vulgaxanthin III), phenylalanine-isobx, tryptophan-bx, tyramine-bx (miraxanthin III), 3-methoxytyramine-bx, phenylalanine-bx, threonine-bx, aspartic acid-bx (miraxanthin II) and γ-aminobutyric acid-bx.

Several factors have been evaluated (some constituents of culture media, light, pH, type and inoculum cultures, utilization of adsorbents) in order to enhance the production of betalains by Beta tissue cultures [40,44], particularly hairy roots [39,41,42,43,45,139,140,141,142].

Several factors may influence the amounts of betalain pigments in the red beetroot such as water stress, growth of plants in the greenhouse or field, different radiation sources either in the nature or in cell cultures, thermal treatment either in industrial or home processing [46,134,135,142,143,144,145,146,147]. In some cases, the biological activities were affected by these factors. For example, the capacity for scavenging free radicals (DPPH, ABTS) and the reductive power are strongly affected by beet root processing [144]. The gamma ray radiation at 30 kGy also influenced the antioxidant activity of betalain extracts, decreasing it. The capacity for inhibiting tyrosinase activity presented a similar behaviour of that reported for the capacity for scavenging DPPH free radicals [134].

Antioxidant activity of betalains from red beet is largely reported, using diverse methodologies for its evaluation. Generally, betacyanins have higher antioxidant activity than betaxanthins. Glycosylation of betalains reduces their ability for scavenging free radicals and consequently decrease their antioxidant activity. The position of glycosylation also affects the radical scavenging property (6-*O*-glycosylated betacyanins exhibit higher free radical scavenging activity than 5-*O*-glycosylated counterparts). The antioxidant activity of betaxanthins is function of the number of hydroxyl and imino residues. Betaxanthins without phenolic hydroxyl groups present only moderate radical scavenging ability [6,26,60,148].

The pH is also an important factor influencing the capacity of betalains for scavenging free radicals. At higher values of pH, the antioxidant activity of betalains generally increases, in contrast to their stability that decreases [7,14]. The encapsulation of some betalains has been made for improving the stability, not affecting their capacity for scavenging free radicals [14,149].

For preventing losses of betalains and respective antioxidant activity, due to thermal treatment of juices, Slavov et al. [150] treated the pressed red beet juice with microwave irradiation. This procedure was able to improve its capacity for scavenging peroxyl radicals. In this case, the amounts of betacyanins decreased in contrast to those of betaxanthins and polyphenols. According to the authors, the best extraction of polyphenols using microwave irradiation may partly explain the best activity found. In addition, the highest amounts of betaxanthins could be result of the synthesis of these pigments during the microwave pre-treatment [150].

Beyond the capacity for scavenging DPPH, ABTS, peroxyl radicals, some authors [151,152] also showed that beet root pomace extracts were able to scavenge other free radicals, such as hydroxyl and superoxide anion radical, being the activities found dose-dependent. A correlation was found between the activities and all phytochemical components (phenols, such as ferulic acid, betacyanins and betaxanthins) detected in the extracts [151,152].

Synergistic antioxidant activity between phenolic compounds and betalains in *B. vulgaris* root extracts was reported [31]. Even in the same organ (root), different antioxidant activities (outer layer had higher activity than the inner part) were found [136]. The harvesting season has also influenced the capacity of red beet root to scavenge the DPPH free radicals. Fresh spring red beet had higher amounts of betalains and antioxidant activity than those samples collected in autumn [153].

A powder derived from beetroot juice, obtained by spray drying, showed stability and antioxidant activity but dependent on the water activity at which they are stored. The greatest stability of the powder was verified for storage atmospheres of a_w_ (water activity) lower than 0.521, whereas the best antioxidant activity was observed for a_w_ values higher than 0.748. However, the powder cannot be stored at this atmosphere a_w_ because rapidly the powder would dissolve [154]. For this reason, the antioxidant activity must be sacrificed in detriment of the stability of the powder.

Beyond the importance of this species for obtaining natural dyes, many properties have been attributed to the beet root betalains, which can prevent some degenerative illnesses, coronary diseases and cancer if consumed in a balanced diet [68].

### 3.5. Celosia argentea *L.*

The genus *Celosia* consists of about 60 species worldwide and is native in subtropical and temperate zones of Africa, South America and South East Asia [155]. There has been a great controversy in the designations of *Celosia argentea*, *Celosia argentea* var. *cristata* and *Celosia cristata*. Some consider *Celosia cristata* as a synonymous of *Celosia argentea*, others as a variety of *Celosia argentea* (var. *cristata* (L.) Kuntze), or a separate species [156]. However, several biometric, cytological and molecular studies have revealed that *Celosia cristata* and *Celosia argentea* can be considered as two species, being *C. argentea* the direct progenitor of *Celosia cristata* [157,158,159,160,161,162,163].

*C. argentea* is considered a wild form, whereas *C. cristata* and *C. plumosa* are considered cultivars. Such designation is based on the flower head form. *C. argentea* has cylindrical pink or rose flower heads with longer bloom stalk; *C. cristata* has crested tightly grouped flowers that form into a wide cockscomb, being these flowers known as brain celosia, wool-flowers or cockscombs; and *C. plumosa* that has feathery plume like flower heads [164,165,166]. In the present work, *C. cristata* and *C. plumosa* are considered species.

The name *Celosia* is derived from the Greek word “kelos”—which means “burned, burning”—owing to the colour of the inflorescences (yellow, red and orange), particularly for *C. plumosa,* which resembles flames erupting from the stems [165,166].

*Celosia plumosa* and *C. cristata* are largely used as ornamental plants but the seedlings, young leaves and inflorescences are also utilised as vegetables in Asia, Africa and South America. Green leaves possess a characteristic sweet taste appreciated in soups [167,168,169,170]. In folk medicine, dried leaves, inflorescences and seeds (semem celosiae) of *C. argentea* are used as disinfectant and ailments for eyes (to improve eyesight) and the liver, in the treatment of dysentery, dysuria, blood and gynaecologic diseases, hypertension, sarcoptidosis, among other disorders [167,171,172,173,174].

Beyond those attributes and applications, *C. argentea* is also considered a weed that may affect the growth of many crops since it inhibits germination and seedling growth of associated crops, such as *Sorghum bicolor*, *Phaseolus aureus*, *Arachis hypogaea*, *Dolichos lablab* and *Vigna unguiculata* [175]. Perveen et al. [176] reported on the possibility of *C. argentea* to be a substitute of herbicides to control some weeds, such as *Lepidium sativum*, that attack crops, due to the results obtained with phenolic-rich extracts of *C. argentea* that were able to control the growth of *L. sativum*.

Laboratorial research has demonstrated diverse biological properties of extracts either in vitro or in vivo conditions: antimicrobial [177,178], anti-inflammatory [179], immunomodulatory [180,181,182,183], antidiabetic [184], antimetastatic [182], anticancer [172,185], healing effects on burn wounds [186,187], inhibitor of α-amylase and α-glucosidase [188]; inhibitor of acetylcholinesterase and tyrosinase [189]; hepatoprotective [174,180,181,190] and antioxidant [168,169,191]. However, there are also reports of the pro-oxidant activity of aqueous and methanolic extracts of *C. argentea*, due to the possible interaction between the molecules with other antioxidants (vitamin C) and minerals (copper, iron) also present in the extract, generating cuprous and ferrous ions that are potent pro-oxidants [192,193]. The biological properties found were generally attributed to phenols [172,183,185] including flavonoids [179], polysaccharides [180,181], saponins [174,190] and bicyclic peptides [194].

The seeds of *C. argentea* (Semen celosiae) are a Chinese herbal medicine for eye and hepatic diseases [195]. The dried ripe seeds are known in China as “Qing Xiang Zi” and are used for the treatment of some diseases such as hepatitis, hypertension, sarcoptidosis and to improve eyesight [173,196]. Saponins (celosins), phenol glycosides and flavonoids may explain some of those properties [67,190,196]. In the seeds, there are also the bicyclic peptides (celogentins and moroidin) that exhibit a huge tubulin polymerization inhibition, with the exception of celogentin K [194,195,197,198,199,200].

**Figure 9:**
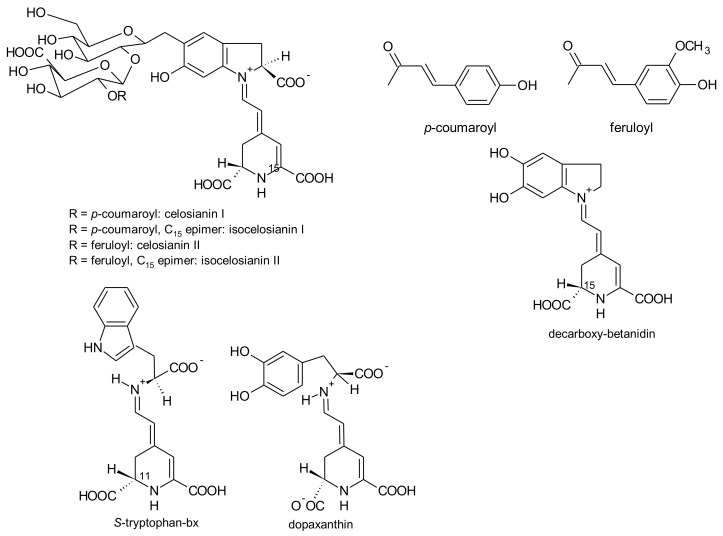
Celosianins, dihydroxylated betalains and *S*-tryptophan-bx from *Celosia*.

The colour of *Celosia* inflorescences may vary from yellow to various shades of red and violet, particularly for the garden *Celosia* (*cristata* and *plumosa*). The pigments involved in these colours are betalains [155]. Six pigments have been reported: amaranthin, isoamaranthin, betalamic acid, dopamine-bx, 3-methoxytyramine-bx and *S*-tryptophan-bx (Figure 4, Figure 6, Figure 7 and Figure 9). However, the amounts of these pigments were different according to the colour of the inflorescences and species. Thus, for the species *cristata*, in the yellow inflorescences predominated dopamine-bx; in the orange inflorescences, amaranthin/isoamaranthin and dopamine-bx were found in similar amounts; whereas in red inflorescences, amaranthin/isoamaranthin were the main pigments. In addition, the amounts of total betalains were also dependent on the colour of the inflorescences. By ascending order, the total amounts of betalains in the inflorescences of the species *cristata* were: yellow < orange < red [155]. In the cultivated *C. plumosa*, dopamine-bx predominated in the yellow inflorescences (Figure 9), such as in the species *cristata*, nevertheless 4 times higher than in this species. In orange-red inflorescences similar amounts of amaranthin/isoamaranthin and dopamine-bx were reported by the authors [155]. Shortly, the red inflorescences of *C. cristata* contained minor amounts of betaxanthins which were in higher amounts in the yellow and orange-red inflorescences. Such results may indicate that there is a gradual reversion of the pigments’ biosynthesis: dopa-*cyclo*-dopa to amaranthin and dopa-dopamine to betaxanthins [155].

The yellow and orange-red inflorescences of the species *plumosa* had always higher amounts of betaxanthins than the yellow and orange inflorescences of the species *cristata* [155]. Cai et al. [167] also described a higher ratio of betaxanthins to betacyanins in the orange-red inflorescences of *C. plumosa* than in the correspondent inflorescences’ colour of the *C. cristata*.

Schliemann et al. [155] did not find celosianins (Figure 9), amaranthin-type (substituted at C-5 of betanidin) betacyanins with acyl groups (*p*-coumaroyl and feruloyl groups) which were already reported by Piatelli and Minale [201] and, later, also reported by Cai et al. [202] for *C. cristata*. The remaining betacyanins and betaxanthins were also reported for the same species [155].

In the yellow inflorescences of *Celosia*, Schliemann et al. [155] found high levels of dopamine, thousand-fold higher than 3-methoxytyramine, thereby the authors warn the possible toxicological effect of these inflorescences when consumed as a vegetable or as an herb in traditional Chinese medicine.

Cai et al. [202] studied the chemical stability and colorant properties of the betaxanthins isolated from *C. argentea*. They found that these pigments were bright yellow in the pH range 2.2–7.0, being more stable at pH 5.5. In addition, the betaxanthins of *Celosia* were more stable than betacyanins at 40 °C but as unstable as red betacyanins (betanin and amaranthin) at temperatures higher than 40 °C. Lyophilised betaxanthins had much higher storage stability than the corresponding aqueous solutions at 22 °C, although refrigeration increased the stability.

In cell culture of *Celosia plumosa*, established from hypocotyls, the new betalains dopaxanthin, betanidin and decarboxy-betanidin (Figure 9) were found beyond dopamine-bx [203]. Cell suspension cultures showed increased production of the dihydroxylated betalains dopaxanthin, betanidin and decarboxy-betanidin. There was also secretion of these pigments from the cells to the culture medium reaching maximal values after 8 days of culture. Along with betalains, the precursor molecules betalamic acid and dopamine were also obtained in the same cell cultures [203].

The effect of light and type of light on the biosynthesis of betalains of *Celosia* was studied [204]. The authors found that white and prolonged far-red irradiation induce the accumulation of betalains in that species. They suggested that the accumulation of these pigments induced by the prolonged far-red irradiation is controlled by the photosynthetic system.

Dopa feeding did not induce an accumulation of betaxanthins by *Celosia*. The same behaviour was observed for other species of the *Amarantaceae* family (particularly of the genera *Gomphrena* and *Iresine*), however for some species of *Portulacaceae*, *Chenopodiaceae* and *Aizoaceae*, when dopa had been added to the apical stem parts with single flower buds or inflorescences, a higher accumulation of betaxanthins were observed [205].

The action of phytohormones on the synthesis of betalains was also studied [206]. These authors reported that indolacetic acid, kinetin, abscisic acid and ethrel at lower concentrations stimulated the betacyanin synthesis and depressed the hypocotyl length of dark grown *Celosia cristata* seedlings. The authors concluded that those compounds, at specific concentrations, control de biosynthesis of betalains through ethylene production in plant tissues.

Bodhipadma et al. [207] investigated and compared the inflorescences of *Celosia cristata,* obtained either in vitro or in vivo. The authors detected that both inflorescences have the same shape but the floret size was smaller in those plants obtained in vitro, despite presenting the same number of bracts, tepals, stamens, ovaries and styles. Both in vitro and in vivo pollen had similar shape, size and germination percentage; however, the formation of seeds did not occur in vitro. The plant regeneration of *Celosia* using in vitro methodologies was also reported [171,208,209] and in vitro plants could be obtained from diverse explants (leaf, stem, shoot tips) and *callus*. Higher percentage of regeneration was possible in well-defined conditions and in vitro plants produced lower mean cell areas but higher nuclear areas when compared to in vivo plants. Somaclonal variation did not occur [208].

### 3.6. Chenopodium quinoa *Willd*

The term *Chenopodium* is derived from the Greek words “chenos”, which means “goose”, and “podos”, which means “foot”, because the leaves often resemble goose feet [210]. *Chenopodium quinoa* Willd (quinoa) is a dicotyledonous herbaceous plant and a seed-producing crop native to the Andean regions of Chile, Peru, Ecuador and Bolivia. The seeds are small, round and flat, are the main edible parts of the crop and their colours range from white and yellow to purple and black [211,212,213,214], although the main commercial varieties are white or black [215].

Quinoa was a staple food of the Incas until the Spanish conquest that introduced wheat and barley although the considerable production losses of these crops due to their poor adaptability to the environmental conditions of the Andean region. In contrast, *C. quinoa* is well adapted to the environmental conditions of that region: frequent drought, frost, hail and wind at an elevation of 3500–3900 m above sea level [211,213,216,217,218]. Quinoa is able to survive with salinity levels as high as those present in sea water (electrical conductivity 40 dS/m to 400 mM NaCl) [216,217,219].

Quinoa is a facultative halophyte that comprises wild relatives and domesticated populations. It exhibits great genetic variability which permits to the crop to grow since altitudes from sea level up to 3900 m above sea level, from extreme aridity (annual rainfall 80 mm) to 2000 mm; in soils with a great range of pH; different photoperiods (short-day and day-neutral cultivars); and withstand temperatures between −8 and 38 °C. Moreover, it has high potential to be cultivated outside its native range [214,218,220,221,222].

There has been an increase of the production of quinoa outside the Andean region, such as United States of America, Canada, India, Italy and China. The interest for the production of that crop is due to the high nutritional and functional properties of seeds. They have a high concentration of proteins (≤23%) providing all of the essential amino acids (lysine, threonine and methionine) nutritionally well balanced; lipids in which prevails linoleic and oleic acids; vitamins (vitamin E and folate); and minerals recommended in the human diet [220,223,224]. Quinoa, a pseudocereal, has been considered gluten-free because it contains very little or no prolamin, and therefore is adequate for celiac patients and for people allergic to wheat [212,225,226]. However, some few cultivars had celiactoxic epitopes, that is, such cultivars had been able to activate the adaptive and innate immune responses in some patients with celiac disease [227].

The traditional uses of quinoa in the Andean region includes whole seeds in soups, “quispiño” (cooked quinoa bread made from raw flour and animal fat), “tactte” (small cake made from quinoa flour fried in fat animal), “pesqhe” (salted porridge with sweet quinoa seeds), bread, noodles and biscuits [221]. The quinoa seeds can be fermented for beer or for a traditional alcoholic beverage called “chichi”. The leaves are also used like spinach and the germinated quinoa seedlings can be incorporated in salads. The whole plant is also used to feed cattle, pigs and poultry [29]. More recently, the application of quinoa broadened and in other regions of the world it has been used in the production of bread (sourdough and non-sourdough), Chinese steamed bread, pasta (tagliatelle and spaghetti), snacks, cookies, edible films and food-grade Pickering emulsions [224].

The leaves, stems and grain have folk medicinal uses in cicatrization, inflammation, analgesia against toothache and as a disinfectant of the urinary tract. It is also used in the case of fractures and internal haemorrhaging and as an insect repellent [228].

Beyond the important nutritional properties of quinoa seeds, they also present antinutritional and bitter-tasting saponins, which must be removed by washing the grain or by mechanical abrasion. The amounts of saponins in seeds determine the “bitter” or “sweet” genotypes. “Bitter” variety contains >0.11% of free saponins, whereas or “sweet” variety contains <0.11% of free saponins [212,229,230]. The main structures of saponins include oleanolic acid, hederagenin and phytolaccagenic acid [231,232].

In one study [231], the authors reported that the amount of saponins in quinoa seeds decrease under saline and drought conditions, thereby a deficit of irrigation could be an interesting and sustainable way to reduce the saponin levels, nevertheless the authors did not describe the effect of the same abiotic factors (salt and drought) on the crop yield. According to the authors, the reduction of saponin content would avoid the necessity to eliminate the outer layers of the seeds where vitamins and minerals are distributed.

Other secondary metabolites can be found in quinoa seeds: phytosterols (β-sitosterol, campestrol, brassicasterol and stigmasterol); phytoecdysteroids [20-hydroxyecdysone, makisterone A, 24-*epi*-makisterone A and 24(28)-dehydromakisterone A]; phenolic acids (ferulic, vanillic and *p*-coumaric) and particularly flavonoids (quercetin, acacetin, apigenin, kampferol and respective glycosides) and betalains [29,214,231,232,233].

Only very few works report the presence of betalains in the coloured seeds of quinoa. Until a recent past, the pigments described for coloured seeds of quinoa were anthocyanins [234]. Only in a work of Tang et al. [214] that, for the first time, the authors identified betacyanins (betanin and isobetanin) in the red and black seeds of quinoa from Canada. Later, Escribano et al. [215] reported more betalains than betanin and isobetanin: amaranthin, dopaxanthin, dopamine-bx or miraxanthin V and proline-bx or indicaxanthin, after analysis of 29 extracts obtained from seeds of different colour (white-black) from Peru. Betacyanins predominated in the red-violet seeds, whereas in yellow ones, betaxanthins predominated. These results contrast with those previously reported [233]. The authors did not detect the presence of betacyanins in some Peruvian red seeds of quinoa. Nevertheless, Abderrahim et al. [235] detected and quantified total betalains (betacyanis and betaxanthins) in Peruvian red seeds of quinoa. The presence of betalains was qualitatively confirmed by the change in the initial colour from red to yellow, after treatment with 2 M sodium hydroxide. According to the absorption spectra of the extracts, the authors [235] classified them in two groups, one presenting a similar spectrum to the beet root extract and the second one in which the peak related to the betaxanthins was absent.

Phenolic fractions of *C. quinoa* seeds revealed capacity for scavenging peroxyl and DPPH free radicals, as well as capacity for reducing Fe^3+^. The authors [214] did not assay the betalain extracts in what concerns antioxidant activity. The capacity for scavenging peroxyl radicals and for reducing metal ions was attributed to the relative high levels of HMF (hydroxymethylfurfural) and not to the phenolic compounds. The presence of this sugar degradation product in the extracts is due to the acid hydrolysis method, at relatively high temperature, that the extracts were submitted without previous remotion of free sugars. Other authors [234,236,237] also reported antioxidant activity of seed extracts of quinoa through the capacity for scavenging free radicals (DPPH, ABTS) and reducing power, which activities were related with phenol content and not with betalains.

Escribano et al. [215] evaluated the antioxidant capacity of extracts obtained from diverse coloured seeds of quinoa (white-black) from Peru. The authors found a correlation between the activity, measured through diverse methods and the colour of seeds. The most active samples were those obtained from red-violet and yellow seeds, that is, in those that exhibited the highest amounts of betacyanins (red-violet) and betaxanthins (yellow).

A direct procedure without previous extraction of antioxidants was followed by Abderrahim et al. [235] for determining the antioxidant activity of diverse coloured seeds of quinoa from Peru, using Cu^2+^ oxidizing reagents. The results showed a positive correlation between the phenols/betalains (betacyanins and betaxanthins) content and antioxidant activity.

Other components, like saponins, which are considered to be anti-nutritionals, may possess some interesting biological properties. Yao et al. [238] showed that saponin-rich quinoa extracts were anti-inflammatory by decreasing the production of the inflammatory mediator nitric oxide and by inhibiting the release of the inflammatory cytokines tumour necrosis factor alpha (TNF-α) and interleukin 6 (IL-6) in LPS-stimulated RAW 264.7 macrophages.

In vivo studies demonstrated that quinoa intake may reduce the risk of developing cardiovascular disease and beneficially modulate metabolic parameters by decreasing the levels of triglycerides, cholesterol, LDL, preventing lipid peroxidation and increasing glutathione (GSH) [29,239,240,241].

The phytoecdysteroid 20-hydroxyecdysone was reported to show neuroprotective effects by modulation of GABA_A_ (γ-aminobutyric acid) receptors as well as hypocholesterolemic activity through the increase of cholesterol transformation into bile acids [242]. The same phytoecdysteroid also had anti-obesity and antidiabetic activity detected in rats that were fed with a high fat diet. The authors linked such attributes to a reduction of absorption of lipids and increase of glucose oxidation, mitochondrial oxidative phosphorylation and energy expenditure [241,243,244].

## 4. Conclusions

Natural pigments, namely carotenoids, flavonoids, anthocyanins or betalains, have been used in food and pharmaceutical industries, replacing the synthetic pigments, since they are considered less deleterious for human health. However, particularly anthocyanins possess limitations: weak stability to processing and storage conditions. The weak stability of the pigments to pH or temperature is, however, less important for betalains since they are stable for broader pH range and are also able to regenerate after thermal treatment. Nevertheless, natural resources are not inexhaustible being also highly dependent on the climatic conditions. In addition, the possibility to obtain diverse hue colours with synthetic pigments is generally easier when compared to the natural ones, unless diverse pigments of different groups are mixed.

Therefore, several approaches need to be followed in order to obtain natural pigments in a sustainable way, cheaper and closer to the consumer requirements. Biotechnological production of betalains through plant cell culture from betalain producing species, so far showing weak yield, or microbes must be more deeply studied and even considered as alternatives to the traditional production of betalains. Studies regarding the encapsulation of natural pigments, such as betalains, for enhancing their colour stabilities, are possibilities to take into account. Nevertheless, the utilization of these natural products requires that they do not interfere on the final quality of the product where the pigments are applied.
